# The Quantitative Analyses for the Effects of Two Wheat Varieties With Different Resistance Levels on the Fungicide Control Efficacies to Powdery Mildew

**DOI:** 10.3389/fpls.2022.864192

**Published:** 2022-05-16

**Authors:** Aolin Wang, Yanan Zhao, Meihui Zhang, Junhai Yuan, Wei Liu, Jieru Fan, Xiaoping Hu, Yilin Zhou

**Affiliations:** ^1^State Key Laboratory for Biology of Plant Diseases and Insect Pests, Institute of Plant Protection, Chinese Academy of Agricultural Sciences, Beijing, China; ^2^State Key Laboratory of Crop Stress Biology for Arid Areas and College of Plant Protection, Northwest A&F University, Yangling, China; ^3^College of Agriculture and Forestry Science and Technology, Hebei North University, Zhangjiakou, China

**Keywords:** wheat powdery mildew, resistant variety, control efficacy, chemical control, *Blumeria graminis* f. sp. *tritici*

## Abstract

Effective strategies to reduce the occurrence of wheat powdery mildew include the use of resistant varieties and application of fungicides. However, most studies rarely focus on the quantitative value of fungicide reduction using resistant varieties. To explore how the fungicides performed on different resistant wheat varieties to powdery mildew, field experiments were conducted during wheat growing seasons in 2018/19 and 2019/20 to investigate the control efficacies of enostroburin⋅epoxiconazole 18% SC and triadimefon 20% EC to wheat powdery mildew on a highly resistant wheat variety (“Baofeng104”) and a highly susceptible wheat variety (“Jingshuang16”). The analyses of variance on control efficacies showed that the control efficacies of enostroburin⋅epoxiconazole 18% SC to wheat powdery mildew were mostly significantly higher than triadimefon 20% EC under the same conditions (i.e., varieties, dosages). However, both fungicide and variety resistance made variabilities in the mildew disease index and played a significant role in mildew management. Particularly, the variety resistance made the greatest contribution in mildew-reducing, and the disease index could significantly be reduced on the highly resistant variety even in the absence of fungicide treatment. The control efficacies to mildew on the highly susceptible variety mainly depended on the high efficiency of fungicides whereas the highly resistant variety were mainly by virtue of variety resistance through the comparative analyses of linear regression models. Furthermore, the random-coefficient regression models and quantile models quantificationally expounded that the relationships between active ingredient dosage and disease index or control efficacy varied from the effects of variety, fungicide, and year, particular from variety. Thus, a dosage reference table of enostroburin⋅epoxiconazole 18% SC or triadimefon 20% EC for different resistant wheat varieties were provided; it would be helpful for users to formulate an appropriate dosage of fungicide on mildew management in the field and avoid overusing or superfluous application. Further study needs to consider the effects of fungicide reduction on wheat yields, only then the maximum-economic benefits on mildew management can be determined.

## Introduction

Wheat powdery mildew, caused by *Blumeria graminis* f. sp. *tritici* (*Bgt*), is one of the major wheat diseases in the world (Chen and Duan, 2014; Mwale et al., 2014; 2015; Liu et al., 2018, 2021), and poses a significant threat to wheat production and food security (Li et al., 2012, 2018; Cao et al., 2014; Singh et al., 2016; Jevtić et al., 2017). The disease has existed and maintained a more serious state of occurrence since the late 1970s in China due to the increased use of fertilizer, expansion of irrigated production areas, and the wide use of highly susceptible wheat cultivars in fields. According to National Agro-Tech Extension and Service Center statistic data, the occurrence of wheat powdery mildew was about 6.2–7.2 million ha in China from 2000 to 2018, and the average annual actual loss reached about 0.3 million tons (Huang et al., 2020).

In general, the efforts including appropriate fungicide application and variety resistance are two essential measures in disease-reducing and controlling (Petersen, 2015), and the combinations of fungicides and variety resistance were beneficial to optimize the management of plant diseases (Willyerd et al., 2012; Barickman et al., 2017; Ács et al., 2018). In fact, variety resistance can also be exploited to reduce fungicide input in wheat production (Cheythyrith et al., 2019). For instance, the protection effect of resistance increased exponentially with increasing variety resistance, and variety with a high level of resistance offered the greatest potential for fungicide reduction to control potato late blight (Nrstad et al., 2010). Ács et al. (2018) concluded that fungicide application depending on variety resistance caused significant control efficacies of Fusarium head blight rather than higher active ingredient dosage of fungicide. For wheat powdery mildew, the control efficacies of low active ingredient dosage fungicides against wheat powdery mildew on a highly resistant variety were significantly higher in comparison to highly active ingredient dosage fungicides on a susceptible variety (Wang et al., 2021).

The effects of disease management targeted by fungicides on varieties with different resistance levels appeared to very greatly. Most farmers and basic-level units of agriculture production would be inclined to get the specific reference-information of fungicide application on different varieties, so that, with scientific guidance, the application of fungicide in a reasonable range could minimize fungicide inputs. However, most previous studies were qualitative analyses which only focused on whether the resistant variety reduced the application of fungicide. For the management of wheat powdery mildew, a quantitative study on the fungicide reduction showed that if the same control efficacy was achieved, the active ingredient dosage of fungicide to wheat powdery mildew under low nitrogen fertilizer application level could be reduced about 30 g/ha more than that of normal nitrogen fertilizer application level in the field (Sun et al., 2021). Similarly, the quantitative value of fungicide reduction among different resistant wheat varieties in practice would be particularly important.

However, both the fungicide and variety resistance have been rarely studied simultaneously in mildew management or fungicide reduction, so it would be pertinent to study the application of fungicide on mildew management by using different wheat resistant varieties. Field experiments were conducted in two growing seasons to quantitatively investigate the effects of highly resistant and susceptible wheat varieties on the control efficacies of enostroburin⋅epoxiconazole 18% SC and triadimefon 20% EC to wheat powdery mildew. The purposes of the present study were to quantificationally analyze the effects of two wheat varieties with different resistant levels in mildew-reducing and fungicide reduction, and the factors (i.e., variety, fungicide, and year) that affect the exact relationships between disease index or control efficacy with active ingredient dosage.

## Materials and Methods

### Field Plot Experimental Design and Treatment

Field plots were established at Langfang Experimental Station, Institute of Plant Protection, Chinese Academy of Agricultural Sciences (39.5°N, 116.6°E) in Hebei Province, China, in 2018/19 and 2019/20 growing seasons. “Jingshuang16” (a winter wheat variety highly susceptible to powdery mildew) and “Baofeng104” (a winter wheat variety highly resistant to powdery mildew) were planted into the field previously cultivated with wheat only. Each plot consisted of ten 2-m-length rows, spaced 0.25 m apart, and planted at a seeding rate of 120 kg/ha.

To ensure even occurrence of wheat powdery mildew in the experimental field, the artificial inoculations were conducted using the mixed isolates of *B. graminis* f. sp. *tritici* (*Bgt*), which were mixed with five prevalent isolates of E09, E15, E21, E23-(2), and E31 with the virulence to main resistant genes *Pm*1a, *Pm*1e, *Pm*3a, *Pm*3b, *Pm*3c, *Pm*3f, *Pm*3e, *Pm*4*a*, *Pm*4b, *Pm*5a, *Pm*5b, *Pm*6, *Pm*7, *Pm*8, *Pm*17, *Pm*19, *Pm*25, *Pm*30, *Pm*34, and *Pm*“XBD.” The seedlings (sown in 10 cm pots) with sporulating *Bgt* lesions were maintained in a greenhouse compartment and transplanted to the center of each plot on 19 March 2019 and 14 March 2020. Each plot was set two in pots with *Bgt* spreaders. No other diseases and pests occurred in the field plots during the experimental periods.

In two successive growing seasons, two fungicides (the enostroburin⋅epoxiconazole 18% SC and triadimefon 20% EC) were used on two wheat varieties (one highly resistant variety and one highly susceptible variety), and each fungicide × variety with six active ingredient dosages, thus, there were 24 treatments (two fungicides × two varieties × six active ingredient dosages) in each season (shown in Table 1). The experimental design used a randomized block with three replications, giving a total of 72 plots. After the artificial inoculations, fungicides were applied once at the beginning of wheat anthesis which corresponded to Zadoks growth stage10.5.1 (Zadoks et al., 1974) in each year, the dates were 7 May 2019 and 11 May 2020, respectively. The fungicide applications were treated with 2,000 ml of liquids for each plot by a backpack sprayer; the untreated control plots were sprayed with water.

**TABLE 1:** Treatments of the field experiments in this study.

Treatment	Fungicide	Wheat variety	Active ingredient dosage of fungicide/g⋅(ha)^–1^
1	Enostroburin⋅epoxiconazole 18% SC	Baofeng104	0
2			67.5
3			94.5
4			121.5
5			148.5
6			175.5
7		Jingshuang16	0
8			94.5
9			121.5
10			148.5
11			175.5
12			202.5
13	Triadimefon 20% EC	Baofeng104	0
14			60
15			90
16			120
17			150
18			180
19		Jingshuang16	0
20			90
21			120
22			150
23			180
24			210

### Disease Ratings and Assessments

Powdery mildew was evaluated 15 days after fungicide application at the stage of kernel watery ripe (GS 10.5.4) on 22 May 2019 and 26 May 2020, respectively, by quantifying the disease severity on 100 plants. Five positions in each plot (four at the corners and one at the center) were selected for disease assessment; 20 plants arbitrarily chosen at each position were assessed on a “0-to-9” scale (Saari and Prescott, 1975; Sheng and Duan, 1991). Disease index (*DI*) for a plot was calculated as:


(1) $$\text{D}\,I=\sum_{i=0}^{i=9}\text{i}*{\text{n}}_{i}/9*\sum_{i=0}^{i=9}{\text{n}}_{i}\times 100$$

where n_0_, n_1_,…, n_9_ are the number of plants with mildew severity values of 0, 1,… 9, respectively.

The control efficacies to the mildew index were calculated as:


(2) $${\text{C}}_{t\,r\,e\,a\,t\,m\,e\,n\,t}\left(\%\right)=\text{C}=\left[\left(\text{D}I_{c\,h\,e\,c\,k}-\text{D}I_{t\,r\,e\,a\,t\,m\,e\,n\,t}\right)/\text{D}I_{c\,h\,e\,c\,k}\right]\times 100$$

where *DI*_*check*_ was the disease indexes of untreated control plots of highly susceptible variety “Jingshuang16,” and *DI*_*check*_ was used as the control treatment to calculate the control efficacies of fungicides on both highly susceptible and highly resistant varieties. The control efficacies of fungicides on highly resistant variety were constituted from two aspects: variety resistance and fungicide.

### Data Analysis

Analyses of variance (ANOVA) were conducted using SAS 9.4 (SAS Institute Inc., Cary, NC, United States) software. And the significance of control efficacies was performed by Duncan’s multiple comparisons (significant difference at *P* ≤ 0.05).

In our preliminary data analysis, there was a linear relationship between disease index or control efficacy with fungicide dosage (data not shown). Initially, general linear model (PROC GLM module in SAS) was used to estimate the variability in the mildew disease index accounted for by the factors of variety resistance, fungicide, fungicide dosage, and their interactions. The contribution of each factor to the variability of the disease index variation was determined by the ratio of the factor’s sum square to the total sum square in the ANOVA model, which was calculated by SS_*factor*_/SS_*total*_. The initial analysis suggested large effects of variety resistance and fungicide on the relationships between mildew disease index and active ingredient dosage (*DI*- *dosage*), as well as the relationships between control efficacies and active ingredient dosage (*C*- *dosage*). Thus, linear regression of mildew *DI* and control efficacies with active ingredient dosage was carried to each combination of variety, fungicide, and year, respectively (SAS Institute Inc., Cary, NC, version 9.4), and the effects of variety, fungicide, or year on mildew *DI*/*C*- *dosage* relationships were tested through parallel curve analysis. Both the *Lm* and the *Anova* functions from R package STATS (version 4.0.2) were used to conduct the regression analyses.

Moreover, due to the large effects of variety resistance and fungicide, we fitted random-coefficient regression models to quantitatively analyze the relationships of disease index and control efficacy with active ingredient dosage, fungicide, and year, focusing on the consistency of such relationships (i.e., variability in the slope and intercept estimates). The disease index or control efficacy were performed as the response variable, the active ingredient dosage of fungicide as fixed factors, and the combination of year, variety, and fungicide was regarded as random factors in this study. *Lmer* function from R package LME4 (version 4.0.2) was used to fit the random-coefficient models. Wald Chi-square test, via *Anova* function from R package car, was used to evaluate the significance of fixed and random factors.

In total, we have eight datasets (i.e., combinations of year, variety, and fungicide: variety-fungicide-year) to assess the relationships of disease indexes or control efficacies with active ingredient dosage. For each dataset, there were 18 experimental plots, which included six treatments (i.e., six different active ingredient dosages of fungicide application), and each treatment contained three replications. The random-coefficient model took the form of:


$${\text{y}}_{i\,j}=\mu_{i}+{\text{x}}_{i\,j}\,\beta_{i}+\varepsilon_{i\,j},$$

i=1,2,…,8;j=1,2,…,18.

(3) $$\mu_{i}∼\left(\mu ,\sigma_{\mu }^{2}\right),\beta \,\text{i}∼\left(\beta ,\sigma_{\beta }^{2}\right),\varepsilon_{i\,j}∼\left(0,\sigma^{2}\right)$$

where *y*_*ij*_ was disease index or control efficacy in the *j*th plot of the *i*th variety-fungicide-year combination and *x*_*ij*_ was the active ingredient dosage of fungicide. BLUPs (best linear unbiased predictors) of individual intercepts (*μ_*i*_*), slopes (*β_*i*_*) and the residual (*ε_*ij*_*) were generated by the LME4 package (R version 4.0.2).

The variability of disease indexes or control efficacies based on the 10, 30, 50, 70, and 90% quantile models were analyzed by the quantreg package (R version 4.0.2).

## Results

### Control Efficacies of Two Fungicides to Wheat Powdery Mildew on Different Resistant Wheat Varieties

#### 2018/19 Season

The disease indexes and control efficacies of two fungicides to wheat powdery mildew on highly susceptible and highly resistant varieties in 2018/19 season were shown in Table 2. There were significant differences on the control efficacies of fungicides to wheat powdery mildew between different resistant varieties when the same fungicide and active ingredient dosage were used. The control efficacies of each fungicide to powdery mildew on highly resistant variety (“Baofeng104”) were significantly higher than those on highly susceptible variety (“Jingshuang16”), even without the application of fungicide, the control efficacies (effect of variety resistance) to mildew on highly resistant variety reached 79.72%, and were still significantly higher than that with the use of a high dosage of fungicide (202.5 g/ha enostroburin⋅epoxiconazole 18% SC or triadimefon 20% EC) on highly susceptible variety. In addition, the control efficacies of enostroburin⋅epoxiconazole 18% SC to wheat powdery mildew, no matter whether on highly susceptible or highly resistant varieties, were mostly significantly higher than triadimefon 20% EC when the same dosage of fungicide was applied.

**TABLE 2:** Control efficacies of two fungicides to wheat powdery mildew on different wheat resistant varieties in 2018/19 growing season.

Variety	Fungicide	Active ingredient dosage of fungicide/g⋅(ha)^–1^	Disease index	Control efficacy (%)
				
Baofeng104	Enostroburin⋅epoxiconazole	175.5	1.33	98.28a
	18% SC	148.5	2.44	96.85ab
		121.5	3.85	95.03bc
		94.5	5.11	93.40c
		67.5	6.89	91.10d
		0	15.70	79.72g
Jingshuang16	Enostroburin⋅epoxiconazole	202.5	17.45	77.45h
	18% SC	175.5	27.41	64.59i
		148.5	39.04	49.56k
		121.5	42.59	44.97l
		94.5	61.19	20.94o
		0	77.40	−
Baofeng104	Triadimefon 20% EC	180	2.78	96.41ab
		150	4.89	93.68c
		120	7.89	89.81d
		90	10.26	86.74e
		60	12.11	84.35f
		0	15.70	79.72g
Jingshuang16	Triadimefon 20% EC	210	32.33	58.23j
		180	38.32	50.49k
		150	50.22	35.12m
		120	57.00	26.36n
		90	63.89	17.45p
		0	77.40	−

*Different letters indicate significant differences at the 0.05 level by Duncan’s multiple comparisons.*

The contribution of experimental factors (variety, fungicide, active ingredient dosage, and their interactions in 2018/19 season) to the observed variation in wheat mildew *DI* by multiple factor analysis (ANOVA) were shown in Table 3. The effect of variety, fungicide, and active ingredient dosage (i.e., on the intercept) were significant, and variety effect was the most significant factor on disease index, accounting for 70.19% of the total variability. Meanwhile, the interactions between fungicide × active ingredient dosage, variety × active ingredient dosage, and fungicide × variety × active ingredient dosage were also significant (*P* < 0.0001), suggesting the slope in the *DI*- *dosage* relationship varied with the variety and fungicide.

**TABLE 3:** Multiple factor analysis of mildew under different active ingredient dosages of two fungicides on two varieties in 2018/19 season.

Source of variation	DF	Sum Sq	Mean Sq	*F*-value	*P* (>F)
Variety resistance (A)	1	30662.78	30662.78	21776.00	<0.0001
Fungicide (B)	1	654.98	654.98	465.15	<0.0001
Fungicide dosage (C)	5	8669.15	1733.83	1231.33	<0.0001
A × B	1	160.21	160.21	133.77	<0.0001
B × C	5	186.50	37.30	26.49	<0.0001
A × C	5	3145.42	629.08	446.76	<0.0001
A × B × C	5	136.52	27.30	19.39	<0.0001
Block	2	2.01	1.01	0.72	0.49
Error	46	64.77	1.41		
Total variation	71	43682.34			

*P < 0.05 represent statistical significance.*

#### 2019/20 Season

The disease indexes and control efficacies of two fungicides to wheat powdery mildew on highly susceptible and highly resistant varieties in 2019/20 season were shown in Table 4. The results were almost consistent with those in the 2018/19 season. There were greater differences on control efficacies when the same fungicide and dosage were employed on the highly resistant variety or highly susceptible variety, and the control efficacies to powdery mildew on highly resistant variety without the fungicide input reached 87.56%. There were also some differences in control efficacies between the two fungicides when the dosage and variety were the same.

**TABLE 4:** Control efficacies of two fungicides to wheat powdery mildew on different wheat resistant varieties in 2019/20 season.

Variety	Fungicide	Active ingredient dosage of fungicide/g⋅(ha) ^–1^	Disease index	Control efficacy (%)
Baofeng104	Enostroburin⋅epoxiconazole 18% SC	175.5	2.21	97.44a
		148.5	3.33	96.14ab
		121.5	3.88	95.50ab
		94.5	4.49	94.80abc
		67.5	5.03	94.17bcd
		0	10.74	87.56f
Jingshuang16	Enostroburin⋅epoxiconazole 18% SC	202.5	21.05	75.61g
		175.5	31.67	63.30h
		148.5	40.37	53.22j
		121.5	45.93	46.78k
		94.5	64.07	25.76m
		0	86.30	−
Baofeng104	Triadimefon 20% EC	180	2.78	96.78ab
		150	3.27	96.21ab
		120	6.02	93.02cd
		90	7.04	91.84de
		60	8.63	90.00ef
		0	10.74	87.56f
Jingshuang16	Triadimefon 20% EC	210	35.00	59.44i
		180	45.25	47.57k
		150	58.21	32.55l
		120	63.27	26.69m
		90	67.78	21.46n
		0	86.30	−

*Different letters indicate significant differences at the 0.05 level by Duncan’s multiple comparisons.*

The effect of variety resistance (i.e., on the intercept) remained equally the most significant factor on *DI*, contributing 73.98% of the total variability according to multiple factor analysis (Table 5).

**TABLE 5:** Multiple factor analysis of mildew under different active ingredient dosages of two fungicides on two varieties in 2019/20 season.

Source of variation	DF	Sum Sq	Mean Sq	*F*-value	*P* (>F)
					
Variety resistance (A)	1	41620.45	41620.45	2689.80	<0.0001
Fungicide (B)	1	706.82	706.82	453.26	<0.0001
Fungicide dosage (C)	5	8302.90	1660.58	1064.88	<0.0001
A × B	1	415.15	415.15	266.22	<0.0001
B × C	5	224.16	44.83	28.75	<0.0001
A × C	5	4699.40	939.88	602.71	<0.0001
A × B × C	5	207.86	41.57	26.66	<0.0001
Block	2	12.10	6.05	3.88	0.0277
Error	46	71.73	1.56		
Total variation	71	56260.58			

*P < 0.05 represent statistical significance.*

### Relationships Between Mildew Indexes or Control Efficacies and Active Ingredient Dosages (*DI*/*C*-*Dosage*) of Fungicide on Two Varieties

Because of the significant interaction between variety resistance and fungicide dosage as well as considerable fungicide variability, we fitted regression models of *DI*/*C* with active ingredient dosage for each combination of variety, fungicide, and season, respectively, and the relationship models between disease indexes or control efficacies and active ingredient dosages of two fungicides on different resistant varieties in two growing seasons were shown in Tables 6, 7. All the linear relationship models were statistically significant (*P* < 0.0001).

**TABLE 6:** The relationship models between disease indexes and active ingredient dosages of two fungicides on different varieties in two growing seasons.

Variety	Fungicide	Model	
		
		2018/19 season	2019/20 season
Baofeng104	Enostroburin⋅epoxiconazole	y = −0.0799x+14.0	y = −0.0453x+9.5
	18% SC	*R*^2^ = 0.923, *F* = 192, *P* < 0.0001	*R*^2^ = 0.871, *F* = 108, *P* < 0.0001
	Triadimefon	y = −0.0730x+16.2	y = −0.0468x+11.1
	20% EC	*R*^2^ = 0.977, *F* = 665, *P* < 0.0001	*R*^2^ = 0.936, *F* = 234, *P* < 0.0001
Jingshuang16	Enostroburin⋅epoxiconazole	y = −0.300x+81.3	y = −0.326x+88.5
	18% SC	*R*^2^ = 0.954, *F* = 332, *P* < 0.0001	*R*^2^ = 0.973, *F* = 585, *P* < 0.0001
	Triadimefon	y = −0.219x+80.6	y = −0.236x+88.8
	20% EC	*R*^2^ = 0.958, *F* = 362, *P* < 0.0001	*R*^2^ = 0.961, *F* = 391, *P* < 0.0001

**TABLE 7:** The relationship models between control efficacies and active ingredient dosages of two fungicides on different varieties in two growing seasons.

Variety	Fungicide	Model	
		
		2018/19 season	2019/20 season
Baofeng104	Enostroburin⋅epoxiconazole	y = 0.103x+81.9	y = 0.0524x+89.0
	18% SC	*R*^2^ = 0.923, *F* = 191, *P* < 0.0001	*R*^2^ = 0.873, *F* = 110, *P* < 0.0001
	Triadimefon	y = 0.0943x+79.0	y = 0.0542x+87.1
	20% EC	*R*^2^ = 0.974, *F* = 598, *P* < 0.0001	*R*^2^ = 0.939, *F* = 246, *P* < 0.0001
Jingshuang16	Enostroburin⋅epoxiconazole	y = 0.489x−21.1	y = 0.414x−8.2
	18% SC	*R*^2^ = 0.961, *F* = 319, *P* < 0.0001	*R*^2^ = 0.961, *F* = 319, *P* < 0.0001
	Triadimefon	y = 0.352x−15.3	y = 0.323x−10.9
	20% EC	*R*^2^ = 0.977, *F* = 556, *P* < 0.0001	*R*^2^ = 0.947, *F* = 232, *P* < 0.0001

Parallel curve analyses showed that there were significant differences in slopes and intercepts (*P* < 0.0001) in the fitted models of mildew index or control efficacy between different resistant varieties when both the season and fungicide variables were constant. There were also significant differences in the slopes and intercepts (*P* < 0.001) between different seasons when the same fungicide was used on highly resistance variety, while there no significant difference between two growing seasons on highly susceptible variety in most cases. In addition, there were significant differences in the slopes (*P* < 0.01) but no significant differences in intercepts (*P* > 0.05) in the *DI*/*C*- dosage relationship models between two fungicides on highly susceptible variety within the same growing seasons, while there no significant difference in both slopes and intercepts in most cases on highly resistance variety (Tables 8, 9).

**TABLE 8:** Comparative analyses of the relationship models between disease indexes and active ingredient dosages of two fungicides on different resistant varieties.

Model comparison*^a^*	Disease index*^b^*	
	
	Slope	Intercept
2019V1F1∼2020V1F1	χ^2^ = 14.957, *P* = 0.0006	χ^2^ = 15.202, *P* = 0.0005
2019V1F2∼2020V1F2	χ^2^ = 26.616, *P* < 0.0001	χ^2^ = 48.228, *P* < 0.0001
2019V2F1∼2020V2F1	χ^2^ = 1.182, *P* = 0.5538	χ^2^ = 3.930, *P* = 0.1401
2019V2F2∼2020V2F2	χ^2^ = 1.027, *P* = 0.5983	χ^2^ = 9.919, *P* = 0.0070
2019V1F1∼2019V1F2	χ^2^ = 1.178, *P* = 0.5548	χ^2^ = 8.418, *P* = 0.0149
2019V2F1∼2019V2F2	χ^2^ = 13.495, *P* = 0.0012	χ^2^ = 0.069, *P* = 0.9663
2020V1F1∼2020V1F2	χ^2^ = 0.083, *P* = 0.9594	χ^2^ = 5.494, *P* = 0.0641
2020V2F1∼2020V2F2	χ^2^ = 19.014, *P* < 0.0001	χ^2^ = 0.014, *P* = 0.9931
2019V1F1∼2019V2F1	χ^2^ = 57.331, *P* < 0.0001	χ^2^ = 110.084, *P* < 0.0001
2019V1F2∼2019V2F2	χ^2^ = 131.882, *P* < 0.0001	χ^2^ = 55.668, *P* < 0.0001
2020V1F1∼2020V2F1	χ^2^ = 134.208, *P* < 0.0001	χ^2^ = 83.867, *P* < 0.0001
2020V1F2∼2020V2F2	χ^2^ = 67.959, *P* < 0.0001	χ^2^ = 141.637, *P* < 0.0001

*^a^F1 and F2 represent enostroburin⋅epoxiconazole 18% SC and triadimefon 20% EC, respectively; V1 and V2 represent highly resistant variety (“Baofeng104”) and highly susceptible variety (“Jingshuang16”), respectively. ^b^χ^2^ and P indicate statistics of chi-square and p-value (P ≤ 0.05 represent statistical significance), respectively.*

**TABLE 9:** Comparative analyses of the relationship models between control efficacies and active ingredient dosages of two fungicides on different resistant varieties.

Model comparison*^a^*	Control efficacies*^b^*	
	
	Slope	Intercept
2019V1F1∼2020V1F1	χ^2^ = 21.054, *P* < 0.0001	χ^2^ = 23.292, *P* < 0.0001
2019V1F2∼2020V1F2	χ^2^ = 35.243, *P* < 0.0001	χ^2^ = 61.801, *P* < 0.0001
2019V2F1∼2020V2F1	χ^2^ = 10.945, *P* = 0.0042	χ^2^ = 12.196, *P* = 0.0022
2019V2F2∼2020V2F2	χ^2^ = 1.908, *P* = 0.3852	χ^2^ = 5.103, *P* = 0.0780
2019V1F1∼2019V1F2	χ^2^ = 1.141, *P* = 0.5652	χ^2^ = 8.254, *P* = 0.0161
2019V2F1∼2019V2F2	χ^2^ = 15.754, *P* = 0.0004	χ^2^ = 1.585, *P* = 0.4526
2020V1F1∼2020V1F2	χ^2^ = 5.643, *P* = 0.0595	χ^2^ = 0.087, *P* = 0.9576
2020V2F1∼2020V2F2	χ^2^ = 7.772, *P* = 0.0205	χ^2^ = 0.338, *P* = 0.8445
2019V1F1∼2019V2F1	χ^2^ = 69.792, *P* < 0.0001	χ^2^ = 104.624, *P* < 0.0001
2019V1F2∼2019V2F2	χ^2^ = 82.355, *P* < 0.0001	χ^2^ = 136.802, *P* < 0.0001
2020V1F1∼2020V2F1	χ^2^ = 78.288, *P* < 0.0001	χ^2^ = 113.898, *P* < 0.0001
2020V1F2∼2020V2F2	χ^2^ = 67.811, *P* < 0.0001	χ^2^ = 120.981, *P* < 0.0001

*^a^F1 and F2 represent enostroburin⋅epoxiconazole 18% SC and triadimefon 20% EC, respectively; V1 and V2 represent highly resistant variety (“Baofeng104”) and highly susceptible variety (“Jingshuang16”), respectively. ^b^χ^2^ and P indicate statistics of chi-square and p-value (P ≤ 0.05 represent statistical significance), respectively.*

### Effect of Variety-Fungicide-Year Combinations on Control Efficacies of Fungicides to Wheat Powdery Mildew

#### Modeling Relationships of Disease Indexes With Dosage Variables

**FIGURE 1:**
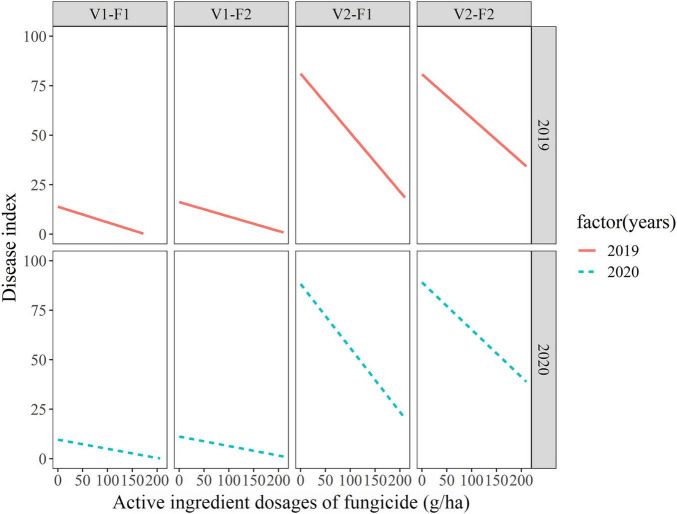
Fitted random-coefficient models relating active ingredient dosages of fungicides to wheat powdery mildew index among different variety-fungicide-year combinations. F1 and F2 represent enostroburin⋅epoxiconazole 18% SC and triadimefon 20% EC, respectively; V1 and V2 represent highly resistant variety (“Baofeng104”) and highly susceptible variety (“Jingshuang16”), respectively.

**FIGURE 2:**
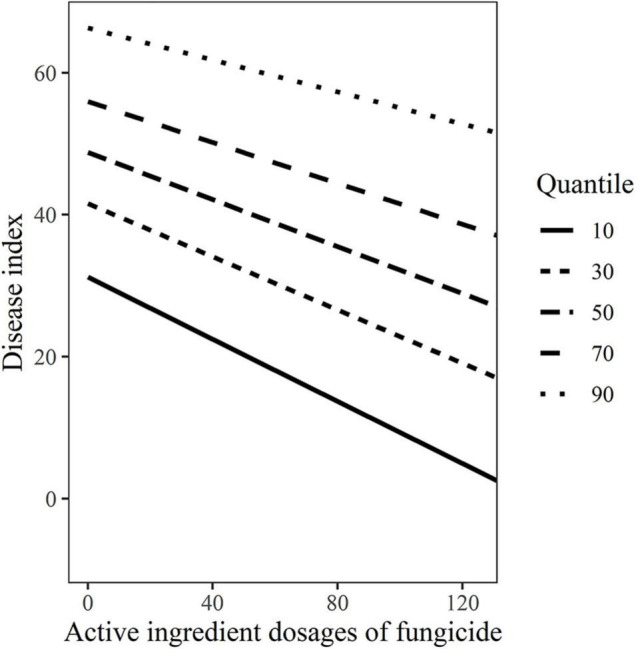
The variability disease index of wheat powdery mildew with active ingredient dosages of fungicides based on the10, 30, 50, 70, and 90% quantile models.

Based on the above results, active ingredient dosages of fungicides were used as fixed effects and variety-fungicide-year combinations were used as random effects in random-coefficient regression modeling of disease index. The fitted model was as follows: *DI*_*ij*_ = *μ_*i*_* + *(dosages_*ij*_)β_*i*_* + *ε_*ij*_*, *μ_*i*_* ∼ (48.76 ± 13.68, 1496.20), *β_*i*_* ∼ (−0.166 ± 0.04, 0.014), *ε_*ij*_* ∼ (0, 7.74). All the three estimates of random-coefficient regression models were significantly different from zero. The models were shown in Figure 1, which indicated considerable differences in the intercepts and slopes, particularly between varieties. The variability of disease index with active ingredient dosages of fungicide based on the 10, 30, 50, 70, and 90% quantile models were shown in Figure 2, which graphically described the variability in the relationships.

#### Modeling Relationships of Control Efficacies With Dosage Variables

**FIGURE 3:**
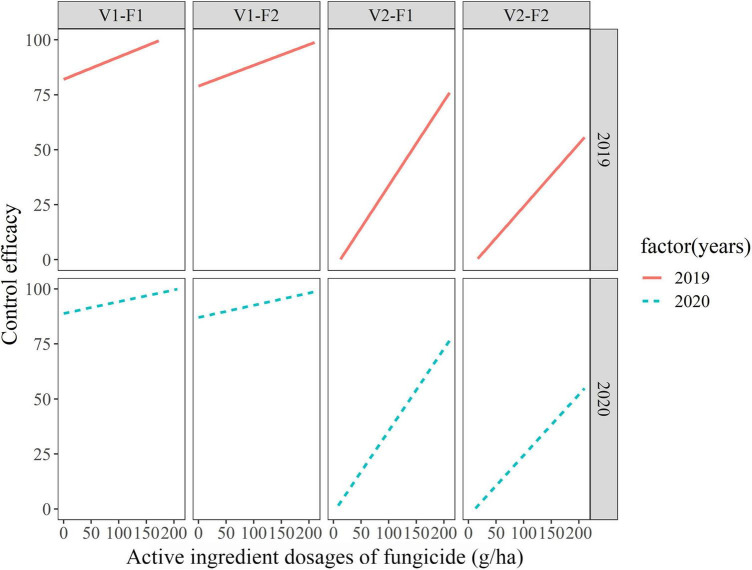
Fitted random-coefficient models relating active ingredient dosages of fungicides to control efficacies on different variety-fungicide combinations among different years. F1 and F2 represent enostroburin⋅epoxiconazole 18% SC and triadimefon 20% EC, respectively; V1 and V2 represent highly resistant variety (“Baofeng104”) and highly susceptible variety (“Jingshuang16”), respectively.

Similarly, active ingredient dosage of fungicide and variety-fungicide-year combinations were used as fixed and random effects in random-coefficient regression modeling of control efficacies, respectively. The variance for the intercept and slope were significantly greater than 0, thus the random effects in the models were significant. The corresponding values for the intercept (μ_*i*_ and σ^2^_μ_) were 40.39 ± 16.56 and 2190.7 ± 46.81, respectively; for the slope, β and σ^2^_β_ were estimated to be 0.20 ± 0.05 and 10.59 ± 3.25, respectively, the variance of residual was 10.59. Modeling relationships of control efficacies also showed considerable differences on intercepts and slopes (Figure 3). As for control efficaciy, there also appeared to be greater variability in the *C*-dosage relationships among varieties than among seasons or fungicides.

**FIGURE 4:**
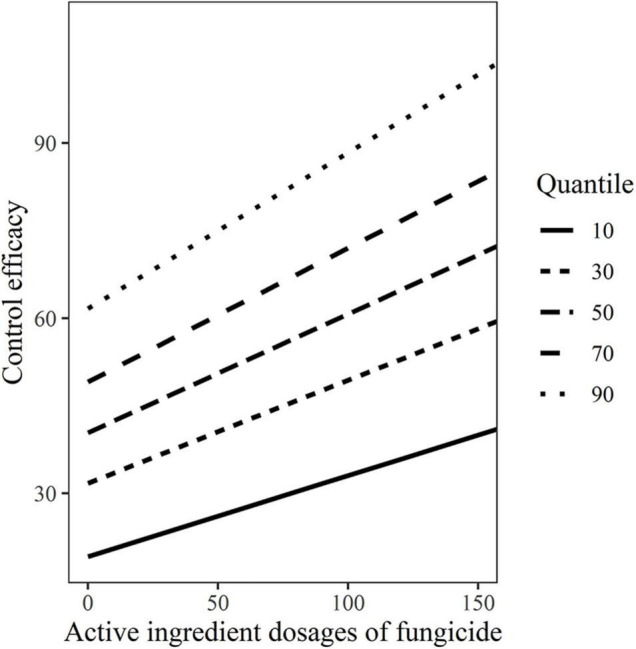
The variability control efficacies with active ingredient dosages of fungicides based on the10, 30, 50, 70, and 90% quantile models.

The variability control efficacies estimation with dosage based on the 10, 30, 50, 70, and 90% quantile models were shown in Figure 4, which also graphically illustrated the variability between control efficacies and dosages.

### Quantitative Study of the Fungicide Application to Wheat Powdery Mildew

The reference table for the active ingredient dosages of enostroburin⋅epoxiconazole 18% SC (Table 10) or triadimefon 20% EC (Table 11) to wheat powdery mildew on different resistant varieties were calculated based on the models of control efficacies or corresponding *DI* (Tables 6, 7). It was obvious that the control efficacies to wheat powdery mildew on highly resistant variety “Baofeng104” (effect of variety resistance) were large enough even without fungicide input, and the quantity demand for active ingredient dosage of fungicide was lower when enostroburin⋅epoxiconazole 18% SC was used on mildew management. Thus, relevant agricultural practitioners could refer to the tables that the specific dosage of fungicide would be reasonably applied on different varieties for mildew management in the field.

**TABLE 10:** Reference table for the active ingredient dosages of enostroburin⋅epoxiconazole 18% SC to wheat powdery mildew on different resistant varieties.

Active ingredient dosage of fungicide/g⋅(ha)^–1^	Highly resistant variety		Highly susceptible variety	
		
	Disease index	Control efficacy (%)	Disease index	Control efficacy (%)
0	9.53∼13.97	81.95∼88.96	81.33∼88.54	−
30	8.17∼11.58	85.05∼90.53	72.33∼78.77	0.00∼4.28
60	6.81∼9.18	88.15∼92.10	63.33∼69.00	8.19∼16.71
90	5.45∼9.18	91.23∼93.67	59.22∼60.31	22.85∼29.14
120	4.10∼4.39	94.35∼95.24	45.33∼49.45	37.51∼41.57
150	1.99∼2.74	96.82∼97.43	36.30∼39.67	52.18∼53.99
180	0.00∼1.38	98.38∼100.00	27.33∼29.90	66.43∼66.84
210	0.00∼0.02	99.95∼100.00	18.33∼20.12	78.86∼81.50
240	−	−	9.33∼10.35	91.29∼96.16

**TABLE 11:** Reference table for the active ingredient dosages of triadimefon 20% EC to wheat powdery mildew on different resistant varieties.

Active ingredient dosage of fungicide/g⋅(ha)^–1^	Highly resistant variety		Highly susceptible variety	
		
	Disease index	Control efficacy (%)	Disease index	Control efficacy (%)
0	11.09∼16.24	79.02∼87.15	80.60∼88.84	−
30	9.69∼14.05	81.85∼88.78	74.02∼81.75	−
60	8.29∼11.86	84.68∼90.43	67.44∼74.66	5.83∼8.49
90	6.89∼9.67	87.51∼92.06	60.86∼67.57	16.40∼18.18
120	5.49∼7.48	90.34∼93.69	54.28∼60.48	26.97∼27.87
150	4.09∼5.29	93.17∼95.32	47.70∼53.39	37.54∼37.56
180	2.69∼3.10	96.00∼96.95	41.12∼46.30	47.25∼48.11
210	0.91∼1.29	98.83∼100.00	34.54∼39.21	56.94∼58.68
240	−	−	27.96∼32.12	66.63∼69.25

## Discussion

The use of resistant variety and fungicide application are critical for disease management, however, most studies only focused on a single factor, such as individual application of fungicide (Ma et al., 2010; Yang et al., 2011; Zhou et al., 2011; Gao et al., 2013; Xu et al., 2014; Guo, 2018). In this study, different fungicides and varieties with different resistance levels to wheat powdery mildew were both implemented at GS 10.5.1 in two consecutive seasons to explore the effects of combining variety resistance and fungicide on mildew index or control efficacy. The results indicated that the mildew index on highly resistant variety could be significantly reduced even in the absence of any fungicide, and the control efficacies of each fungicide on highly resistant variety were significantly higher than those on highly susceptible variety. Meanwhile, the enostroburin⋅epoxiconazole 18% SC expressed a greater effect in mildew-reducing, especially on highly susceptible variety, and the active ingredient dosage of enostroburin⋅epoxiconazole 18% SC applied on highly susceptible variety could be reduced about 30% compared with triadimefon 20% EC when the control efficacies or disease index were approximately identical.

Previous studies found that there was an additive effect between the factor of variety resistance and fungicide (Willyerd et al., 2012; Yan, 2019). In our research, the multiple factor analysis of mildew index showed that the effect of fungicide and variety on mildew index were both significant. It meant that the control efficacies of fungicides certainly include the effects of two factors from variety resistance and fungicide, which should both be fully considered in mildew-reducing. Besides, studies have found that the differences on control efficacy to disease depended more on wheat cultivar (Zală et al., 2014), and the high resistance level of wheat variety to mildew was a key factor for mildew-reducing (Zhou et al., 2003); our result was similar to these studies, which showed that the effect of variety resistance on mildew-reducing was greater than the fungicide and other factors, and contributed nearly 80% on the mildew index when the highly resistant variety was used in our experiment.

Because of the significant interaction between variety, fungicide, and active ingredient dosage as well as among-season variability, the *DI*/*C*- *dosage* models were compared under the condition of different varieties/fungicides/years through the parallel curve analyses. There were invariably significant differences both in the slopes and intercepts of models from variety to variety, which indicated that the differences in resistance-level of wheat contributed to the variability of disease-reducing, and the results were consistent with previous studies (Hollingsworth et al., 2008; Paul et al., 2008; Willyerd et al., 2012). Meanwhile, there were also some significant differences in the slopes or/and intercepts on *DI*/*C*- *dosage* models between two fungicides or between 2 years, which suggested that the control efficacies to mildew on highly susceptible variety mainly depend on the control efficiency of two tested fungicides, whereas mildew-reducing achieved on highly resistant variety primarily by virtue of variety resistance and secondarily by fungicide. It means that, if a resistant variety is used in wheat production, the use of fungicide may be only a small amount and even unnecessary. However, the effects of year mainly depended on the severity of disease epidemic and probably had randomness and uncertainty to some extent. Meteorological conditions (Supplementary Table 1) such as higher relative humidity, higher wind speed, and lower solar radiation in 2020 were probably more favorable for powdery mildew epidemic, especially on highly susceptible variety, which was consistent with previous study (Cao et al., 2015).

According to random-coefficient regression models of *DI*/*C*- *dosage* relationships for each combination of variety-fungicide-year, the random effects of variety-fungicide-year combinations were significant, and the exact relationships of mildew index or control efficacy with active ingredient dosage varied significantly from variety-fungicide-year, particularly between varieties. In other words, the specific-quantitative value of control efficacy or mildew index were significantly different by treating with the given active ingredient dosage. Meanwhile, the quantile models based on random-coefficient models achieved a consistent finding. The exact *DI*/*C*- dosage relationships also varied between different quantiles, with increasing dosage, the interval between high quantile and low quantile on *DI*/*C* also grew accordingly (Figures 2, 4). When the recommended active ingredient dosage of fungicide (150 g/ha for each fungicide) was applied for mildew management, the maximum differences on disease index and control efficacy between 90% quantile and 10% quantile were 50.92 and 60.37, respectively. Certainly, except the information of dosage, fungicide, and wheat variety, the largely varied quantile models of disease index or control efficacy contained more comprehensive information, such as some unpredictable factors that varied between 2 years which were derived from natural and artificial conditions (i.e., meteorological/environmental conditions, plant growth vigor/nutrient status, and so on) may also contribute to the variability. Thus, it is critical to clarify the effects of fungicides, resistance level of variety, and growing seasons. In addition, some other findings could be derived from the quantile models. When the 150 g/ha active ingredient dosage of fungicide was applied, the probability of the mildew index limited to 34.31% was 70% (0.7 quantile of *DI*), and the probability of control efficacy more than 58.48% was 70% (0.3 quantile of *C*).

The susceptible varieties to powdery mildew are widely used in wheat production in China (Zhan et al., 2010; Li et al., 2016), consequently, fungicide application is still the most effective means of disease control. However, while the benefits of fungicides are being enjoyed by more investment in mildew management, other effects may cause a series of risks such as increased fungicide resistance of the pathogens (Pan et al., 2019). To maintain the service-life of fungicides, an economical and practical approach is the use of resistant varieties, which play an essential role in fungicide-reduction, such as the reduction of dosages or the application times (Paparu et al., 2014; Gummert et al., 2015; Pakki and Djaenuddin, 2019). However, there are large variations between different resistant varieties in the requirement for fungicide application to control disease. And most of the disease managements have not formulated a specific scheme of fungicide application on different resistant varieties, particularly in current practice of wheat production. Thus, it is necessary to adjust the dosage of the fungicide application according to the resistance level of varieties (Schepers et al., 1996; Paul et al., 2008; Nrstad et al., 2010), so that the farmers would refrain from overusing fungicides in crop production (Zhang C. et al., 2015), and the risks to the environment posed by fungicides would also be controlled (Zhang M. H. et al., 2015). In the present study, we established reference tables of fungicide application to provide a quantificationally desired value of control efficacy or disease index for a given active ingredient dosage on two different resistant wheat varieties, so that the relevant units of agricultural production and farmers could formulate the corresponding dosage of fungicide application directly based on the reference tables. In practice, the most appropriate dosage of fungicide can be considered for field application on mildew management to achieve a desired performance and the input of fungicide will be reduced drastically. Through this, less investment of fungicide in mildew-reducing, but more economic benefits, could be acquired for farmers.

In conclusion, our results supported that both fungicide and variety resistance played a significant role in mildew management, and the factor of variety resistance made the greatest contribution in mildew-reducing. The control efficacies to mildew on highly susceptible variety mainly depend on the high efficiency of fungicides, whereas on highly resistant variety, mildew-reducing was achieved primarily by virtue of variety resistance and secondarily by fungicide. Furthermore, the random coefficient regression and quantile models quantificationally expounded that the *DI*/*C*- *dosage* relationships varied from variety, fungicide, and year, particularly from variety. Thus, a dosage reference table of fungicide application about the mildew indexes and control efficacies among the different active ingredient dosages of fungicides on highly susceptible or resistant varieties were provided, which would be helpful for users to formulate an appropriate dosage on mildew management in the field and avoiding fungicide overusing or superfluous application. However, the quantitative analysis on fungicide reduction only based on one highly resistant variety and one highly susceptible variety using two fungicides, more kinds of fungicide (mechanisms of action) and different resistance levels of variety (e.g., moderate susceptible and moderate resistant variety) should be considered in future field trials to obtain full and accurate experimental data. And the effects of fungicide reduction on wheat yields should also be quantitatively analyzed in further study; only then the maximum-economic benefits on mildew management can be determined.

## Data Availability Statement

The raw data supporting the conclusions of this article will be made available by the authors, without undue reservation.

## Author Contributions

AW, WL, JF, JY, XH, and YiZ conceived and designed the experiments. AW, WL, YiZ, and MZ performed the experiments. AW, WL, and YiZ analyzed the data. AW, WL, YaZ, JF, XH, and YiZ wrote the manuscript. All authors have read the manuscript and agreed with its content.

## Conflict of Interest

The authors declare that the research was conducted in the absence of any commercial or financial relationships that could be construed as a potential conflict of interest.

## Publisher’s Note

All claims expressed in this article are solely those of the authors and do not necessarily represent those of their affiliated organizations, or those of the publisher, the editors and the reviewers. Any product that may be evaluated in this article, or claim that may be made by its manufacturer, is not guaranteed or endorsed by the publisher.
