# Supplementary material for: The Quantitative Analyses for the Effects of Two Wheat Varieties With Different Resistance Levels on the Fungicide Control Efficacies to Powdery Mildew
Source: Front Plant Sci. 2022 May 16;13:864192. doi: 10.3389/fpls.2022.864192 (PMC9149294; doi:10.3389/fpls.2022.864192)
Supplement: Supplementary file 1 [file Table_1.DOCX]

Supplementary Material

**Table S1.** Meteorological data in the tested field in Langfang City, Hebei Province.

| **Year** | **T/℃** | **RH/%** | **SR/W m^-2^** | **WS/m s^-1^** | **VPD/mbar** | **P/mm** |
| --- | --- | --- | --- | --- | --- | --- |
| 2019 | 17.03 | 58.41 | 0.22 | 1.14 | 1005.67 | 50.50 |
| 2020 | 16.48 | 67.51 | 0.21 | 1.37 | 1005.04 | 9.30 |

Meteorological data were collected using a Dynamet weather station (Dynamax, Inc., Houston, Texas, USA) placed about 20 m away from the experimental field.

The 15-days meteorological data, from the date of fungicide application to the date of disease ratings, were considered: mean temperature of 15 days (T), mean relative humidity of 15 days (RH), mean solar radiation of 15 days (SR), mean wind speed of 15 days (WS), mean vapor pressure deficit of 15 days (VPD), total rainfall of 15 days (P). The raining days were 3 and 5 times in 2019 and 2020 within the period of 15 days, respectively.
